# Cellular transcription factor TFII-I represses adenovirus gene expression

**DOI:** 10.1128/jvi.00618-25

**Published:** 2025-05-12

**Authors:** Rachel L. White, Patrick Hearing

**Affiliations:** 1 Department of Microbiology and Immunology, Renaissance School of Medicine, Stony Brook University 273107, Stony Brook, New York, USA; The University of Arizona, Tucson, Arizona, USA

**Keywords:** adenovirus, TFII-I, GTF2I, gene expression, Viral DNA Replication

## Abstract

To successfully replicate, viruses must overcome innate cellular antiviral responses. With human adenovirus (HAdV), a key viral repressor of cellular antiviral responses is the early protein E4ORF3. E4ORF3 relocalizes numerous cellular antiviral proteins, particularly those involved in the interferon (IFN) and DNA damage response (DDR) pathways, to sequester them from viral replication sites in the nucleus. E4ORF3 also directs SUMO modification of a subset of its targets, some of which are subsequently targeted for proteasomal degradation. We previously identified TFII-I, a cellular transcription factor and DNA repair protein, as one of the proteins most highly SUMOylated by E4ORF3, as well as one of the E4ORF3 degradation targets. In this study, we characterized the effect of TFII-I knockout (KO) on HAdV replication. TFII-I KO significantly increased the infectious virus yield from infected cells, supporting the hypothesis that TFII-I acts as a restriction factor during HAdV infection. TFII-I KO also significantly increased viral genome replication, as well as both early and late gene and protein expression. Our results do not support TFII-I acting as a part of either the DDR or IFN responses during HAdV infection. Our results characterize a novel antiviral function for TFII-I against HAdV that occurs during the early stage of the viral replication cycle and highlight the importance of studying viral countermeasures to the cellular antiviral response, like E4ORF3, to better understand how cells restrict viral infection.

**IMPORTANCE:**

The cellular transcription factor TFII-I was previously shown to bind to HAdV late promoters and to E4-mutant viral genomes during replication. More recently, TFII-I was shown to be a degradation target of HAdV protein E4ORF3. Due to the long-established importance of E4ORF3 in countering cellular antiviral responses, this raised the question of whether TFII-I possesses an undiscovered antiviral role against HAdV. It was hypothesized that whether TFII-I played an antiviral role in HAdV infection, it was most likely to be as a repressor of the late transcriptional program. Here, we show the first direct evidence of TFII-I repressing HAdV infection and demonstrate that the inhibitory effect can be detected much earlier in the viral life cycle than previously predicted. Our findings provide insight into the role of TFII-I in the cellular antiviral response.

## INTRODUCTION

Human adenoviruses (HAdV) are a family of dsDNA viruses that commonly cause mild gastrointestinal, ocular, or respiratory infections in otherwise healthy individuals, as well as more severe infections in the immunocompromised, such as transplant recipients (1). HAdV is a well-established model for studying DNA virus infection and pathogenesis (2). Like most DNA viruses, the HAdV replication cycle has several distinct stages of viral gene expression, including the immediate early, early, and late phases of infection (3). The HAdV early proteins include those required for viral genome replication, as well as ones which function to either reorganize the cell to favor viral replication or to block the cellular antiviral response to infection (2, 4). The highly conserved E4ORF3 protein antagonizes several cellular responses to HAdV infection (4). E4ORF3 multimers form filamentous nuclear structures known as tracks, which can bind and sequester specific cellular proteins away from sites of viral replication in the nucleus (5). The E4ORF3 protein is a SUMO E3 ligase, which directs the SUMO modification (SUMOylation) of a specific subset of the cellular proteins colocalized within nuclear tracks (6). SUMOs (small ubiquitin-like modifiers) are small proteins that are structurally and functionally similar to ubiquitin, able to be reversibly conjugated to substrate proteins to regulate their activity. In some cases, proteins SUMOylated by E4ORF3 are subsequently polyubiquitinated and degraded by the proteasome (7). E4ORF3, therefore, represents a powerful tool for HAdV to block cellular antiviral mechanisms and rearrange the cell nucleus to favor viral replication.

E4ORF3 inhibits multiple cellular antiviral responses, with several of its targets involved in the interferon (IFN) response (8, 9). With species C HAdV, E4ORF3 also targets cellular proteins involved in the DNA damage response (DDR) (10–13). The HAdV linear dsDNA genome resembles a double-strand break (DSB) in cellular genomic DNA. As DSBs are a particularly dangerous form of DNA damage, cells encode multiple proteins that efficiently identify and repair DSBs, such as the Mre11-Rad50-Nbs1 (MRN) complex (13). MRN proteins can bind the termini of the HAdV genome to trigger a DDR (14), which leads to partial degradation and ligation of the HAdV genome into concatemers (15, 16). If not prevented, this process is highly limiting to HAdV replication (10–12, 15, 16). Preventing a DDR response to the viral genome is so vital to successful HAdV infection that species C HAdV encodes two independent methods of inhibiting the MRN complex: as described, E4ORF3 binds and sequesters MRN proteins in nuclear tracks, and the E4ORF6/E1B-55K Ubiquitin ligase complex targets MRN proteins for proteasomal degradation (11, 17). Consequently, E4ORF3^-^/E4ORF6^-^ mutant viruses are replication deficient in cells with a normal DDR response (18, 19). E4ORF3 also interferes with the cellular response to IFNs, signaling proteins that serve a key role in the innate immune response to viruses. For example, E4ORF3 targets several proteins, such as PML and Daxx (8, 9), which are important for the formation of PML nuclear bodies, nuclear structures that are induced in response to IFN signaling and can restrict the replication of multiple DNA viruses (20, 21). Studying how E4ORF3 alters the host cell nucleus to promote viral replication can reveal new insights into innate cellular antiviral defenses that viruses must be able to overcome to replicate.

We previously demonstrated that HAdV-C5 (Ad5) E4ORF3 induces significant SUMOylation of the cellular transcription factor TFII-I, to an even greater degree than well-characterized targets such as the MRN proteins and PML (6). TFII-I is not only relocalized to E4ORF3 to nuclear tracks, but it is also targeted for ubiquitin-mediated proteasomal degradation (7). These results suggest that TFII-I may have a hitherto unknown ability to negatively impact HAdV replication. However, it was unclear which function(s) of TFII-I may relate to its potential antiviral activity. Interestingly, only the E4ORF3 protein of species C HAdV targets TFII-I to nuclear tracks and for degradation (7).

TFII-I is a ubiquitously expressed, multifunctional cellular protein, encoded by the gene GTF2I. TFII-I was first identified as a transcription factor that bound to initiator (Inr) sequences in DNA promoters, but it has since been found to bind other sequences, such as E-box elements (22). TFII-I binding to cellular gene promoters can lead to the activation or repression of genes involved in a broad variety of functions, such as the cell cycle and cellular responses to stress or growth factors. TFII-I has also been shown to be an important accessory protein for other transcription factors, such as CTCF, and to regulate transcriptional elongation (22–26). More recently, TFII-I has also been found to act as a DNA repair protein in translesion synthesis, forming part of the recruitment complex for Pol ζ to DNA lesions (27), and potentially also in homologous recombination (HR) repair of DSBs in DNA (28, 29). With respect to HAdV, a previous study found that TFII-I bound and repressed a promoter on the viral genome, L4P, whose products are required for proper regulation of the transition from the early to late stages of HAdV replication (30).

In our current study, we sought to further understand what role TFII-I may play during HAdV infection. We demonstrate that TFII-I knockout (KO) cell lines have a significant increase in infectious viral titers after infection with wild-type Ad5, confirming that TFII-I acts as an antiviral protein. TFII-I KO also leads to a transient, but significant, increase in viral DNA replication during Ad5 infection, as well as changes in the kinetics of viral early and late gene and protein expression. We did not find a role for TFII-I in the DDR- or IFN-mediated antiviral response to Ad5. Our results are consistent with a mechanism of TFII-I inhibition of viral immediate early gene expression, causing subsequent changes in viral protein, DNA, and infectious virus production.

## RESULTS

### TFII-I does not fit the profile of other E4ORF3 targets

**Fig 1:**
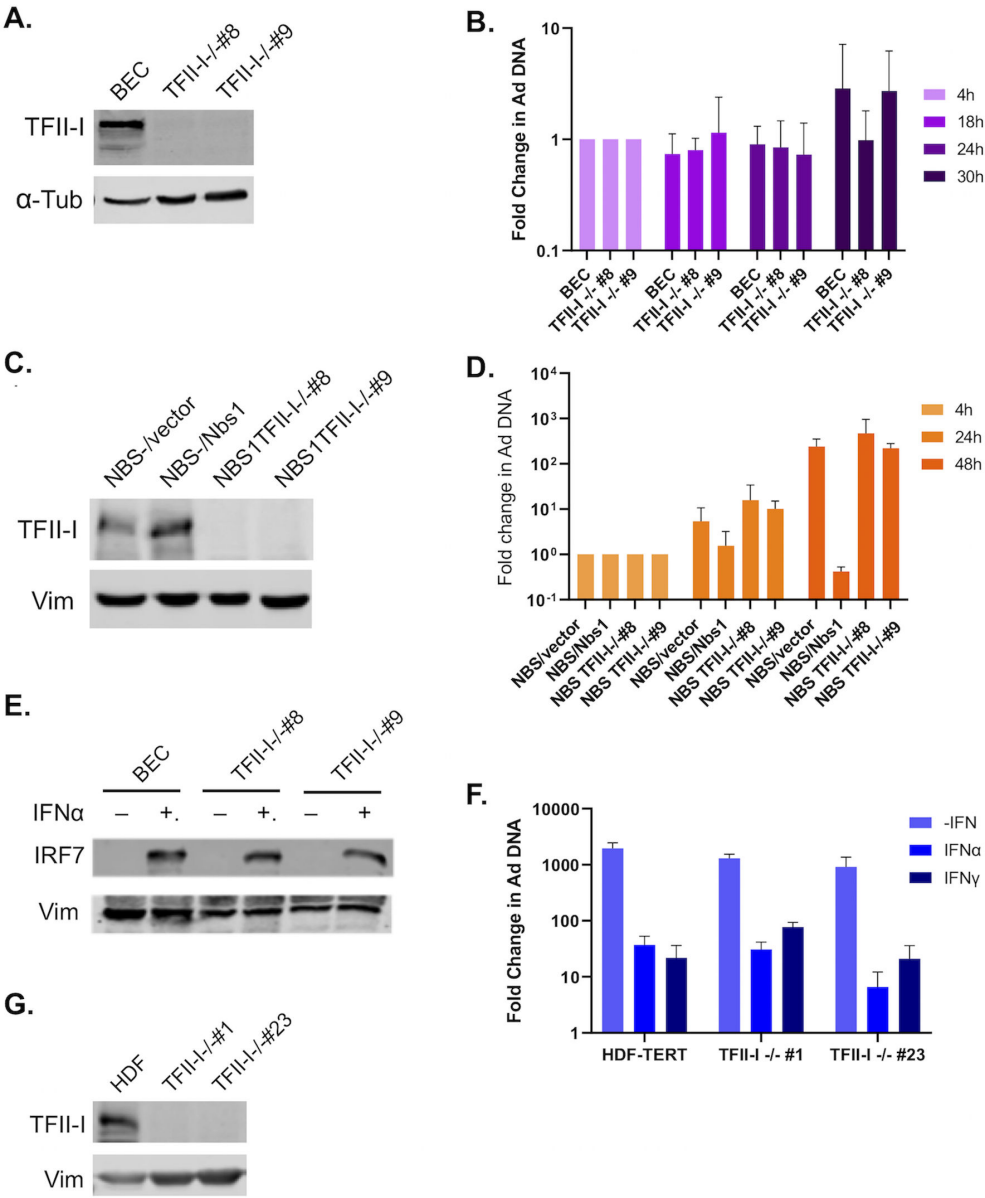
TFII-I KO does not alter the DDR or IFN response to Ad5 infection. TFII-I KO cells were generated by infecting parental BEC, NBS1-, or HDF cells with a CRISPR-Cas9 and guide RNA containing Lentivirus, followed by clonal selection. TFII-I KO was confirmed via Western blot (A, BEC; C, NBS1-; and G, HDF); α-tubulin or vimentin was measured as loading controls. Two independent TFII-I KO clones were selected for each cell type. (**B**) Parental and TFII-I KO BECs were infected with E4ORF3^-^/E4ORF6^-^ Ad5 at an MOI of 1, and total cellular DNA was isolated at the indicated times post-infection. HAdV genome levels were quantified by qPCR and normalized to endogenous GAPDH levels. The results shown represent the mean values ± SD; *n* = 3. (**D**) NBS1^-^ (NBS/vector), NBS1^-^ ectopically expressing Nbs1 (NBS/Nbs1), and NBS1^-^ TFII-I KO cells (NBS TFII-I-/- #8 and #9) were infected with E4ORF3^-^/E4ORF6^-^ Ad5 (dl355/inORF3) at an MOI of 1, and total cellular DNA was isolated at the indicated time post-infection. HAdV genome levels were quantified by qPCR and normalized to endogenous GAPDH levels. The results shown represent the mean values ± SD; *n* = 3. (**E**) Parental and TFII-I KO BECs were pretreated for 24 h with IFNα. IRF7 levels were measured by Western blot; Vimentin was measured as a loading control. (**F**) Parental and TFII-I KO HDFs were pretreated for 24 h with IFN-α or IFN-γ and infected with Ad5-WT at an MOI of 1. Total cellular DNA was isolated at the indicated times post-infection. HAdV genome levels were quantified by qPCR and normalized to endogenous GAPDH levels. The results shown represent the mean values ± SD; *n* = 3.

Many of the cellular proteins targeted by E4ORF3 that are most conserved between HAdV species are part of the PML-mediated IFN response; in addition, species C HAdV E4ORF3 also targets multiple DDR proteins that can inhibit viral DNA replication (4, 5, 8–10, 13, 31, 32). We were therefore interested in examining whether TFII-I plays a role in these antiviral pathways. While TFII-I was first identified and has been most studied as a transcription factor, a few recent studies have found it to also act as a DNA repair protein in translesion synthesis and homologous recombination (HR) of double-strand breaks (DSB) (27–29). While neither of these DDR pathways is known to target HAdV, an alternative DSB repair pathway, non-homologous end joining (NHEJ), is known to be strongly inhibitory to HAdV replication. The MRN complex activates NHEJ of HAdV genomes if not inhibited during viral infection (10–12, 33). We therefore wanted to determine whether TFII-I knockout (KO) rescues replication of an Ad5 E4ORF3^-^/E4ORF6^-^ mutant virus. Toward this end, we generated TFII-I KO cell lines in normal human bronchial epithelial cells (BECs) using a CRISPR-Cas9 strategy. Two independent TFII-I KO cell clones that showed full loss of TFII-I expression were selected for further experimental use (Fig. 1A). We infected parental and TFII-I KO BECs with the E4ORF3^-^/E4ORF6^-^ mutant virus dl355/inORF3 (19) and quantified viral DNA replication using qPCR. Our rationale was based on the observations that mutations in the MRN protein block HAdV-C5 induction of a DDR and partly rescue replication of an E4ORF3^-^/E4ORF6^-^ mutant virus (10, 11). If TFII-I is involved in a DDR to HAdV infection, then TFII-I KO may ablate the effect. The E4ORF3^-^/E4ORF6^-^ mutant virus replicated poorly in BECs, and TFII-I KO did not rescue replication, as there was no significant difference in viral DNA levels between the parental and TFII-I KO cells (Fig. 1B). Although this suggested that TFII-I was unlikely to play a significant role in the MRN-mediated inhibition of HAdV replication, other DDR proteins are also known to have comparatively minor but still repressive roles during HAdV infection (31). We therefore also looked at the effect of TFII-I KO in NBS1^-^ cells (Fig. 1C), which lack the MRN complex but are otherwise normal for DNA damage repair (34). The E4ORF3^-^/E4OF6^-^ mutant virus replicated to a limited extent in NBS1^-^ cells, and this was inhibited when Nbs1 expression was restored (Fig. 1D), as previously reported (34). No significant change in mutant viral DNA replication was observed in NBS1^-^ cells as a result of TFII-I KO compared to the parental cells (Fig. 1D), suggesting that TFII-I is unlikely to play a role in alternative DDR responses to the HAdV genome.

As E4ORF3 is known to block the IFN response to HAdV infection (8, 9), we also explored whether TFII-I might play a role in this antiviral response. To date, TFII-I has not been associated with the cellular IFN response to viruses, but one previous report did find that TFII-I knockdown decreased expression of the IFN-stimulated gene, IRF7, albeit in the absence of IFN (23, 35). However, when we treated the parental BECs and TFII-I KO cells with IFNα, which is known to stimulate IRF7 expression (36), we saw no change in IRF7 expression with TFII-I KO compared to the parental BECs (Fig. 1E). Furthermore, no increase in IRF7 expression was observed with TFII-I KO minus IFN treatment. Pretreatment of normal human diploid fibroblasts (HDFs) with IFN-α or IFN-γ prior to Ad5-WT infection significantly reduces viral DNA replication (37). We were therefore interested in determining whether TFII-I KO relieved this effect, as this could indicate that TFII-I is active in the IFN response to HAdV infection. We generated TFII-I KOs in HDFs (Fig. 1G) and infected IFN-treated parental and TFII-I KO HDFs with Ad5-WT. We found that TFII-I KO did not rescue HAdV replication in IFN-treated cells (Fig. 1F), indicating that TFII-I is unlikely to act as part of the IFN response to HAdV infection.

### TFII-I KO increases viral yield in Ad5-WT infection

**Fig 2:**
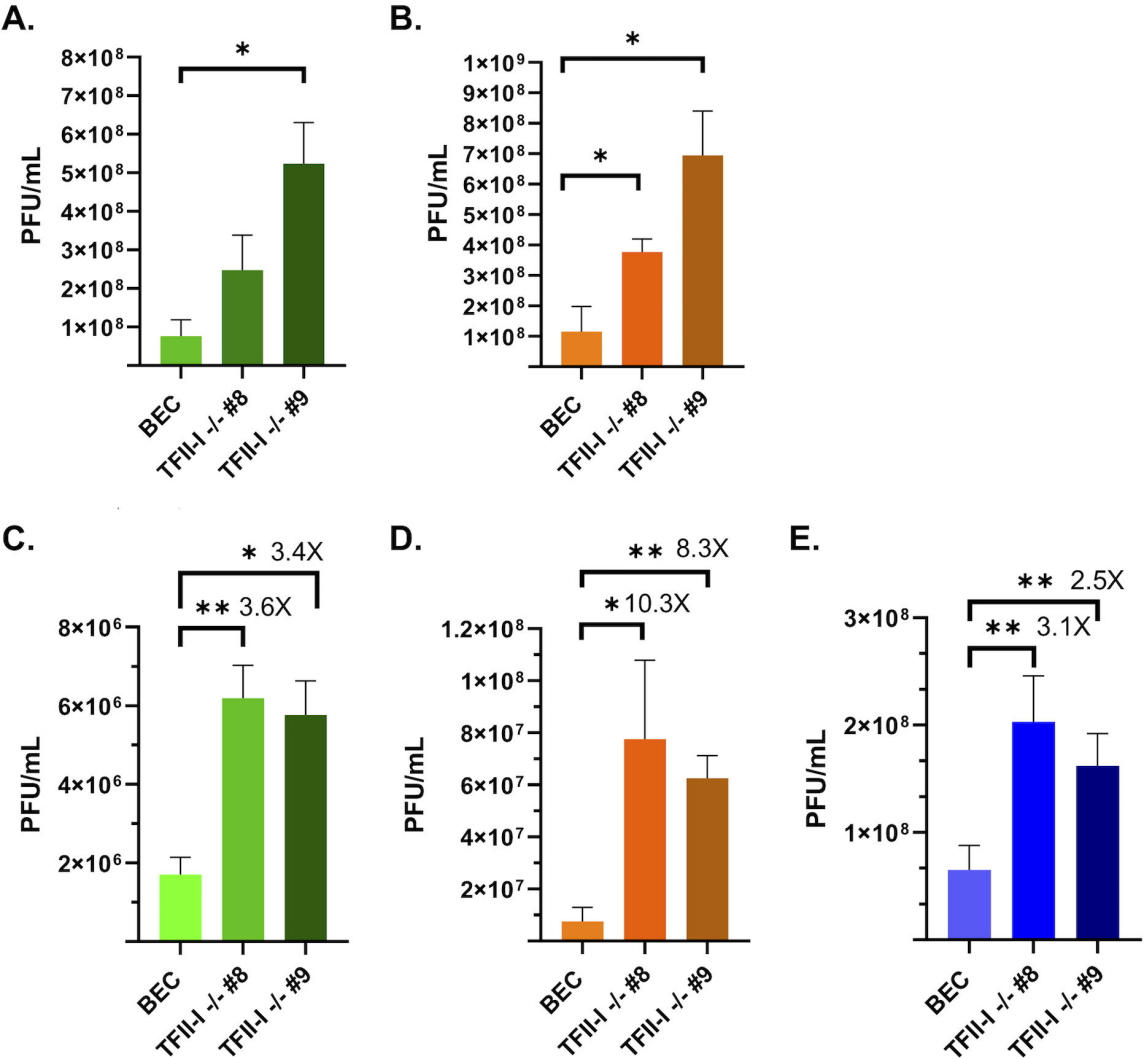
TFII-I KO increases viral yield during Ad5-WT infection. Parental and TFII-I KO BECs were infected with Ad5-WT at an MOI of 1, and cell lysates were collected at 48 (**A**) and 72 (**B**) hpi. Viral yields were determined by plaque assay. To generate a multistep growth curve, parental and TFII-I KO BECs were infected with Ad5-WT at an MOI of 0.1, and cell lysates were collected at 3 (**C**), 6 (**D**), and 9 (**E**) dpi. Viral yields were determined by plaque assay. The results shown represent the mean values ± SD; *n* = 3. * =*P* ≤ 0.05, ** =*P* ≤ 0.01. The fold increases in virus titers in the multistep growth curves at each time point are shown.

As our initial results did not support a role for TFII-I in either the DDR or IFN responses to HAdV, we next wished to determine whether TFII-I KO was beneficial for viral replication. To do so, we infected parental and TFII-I KO BECs with Ad5-WT and quantified the resulting infectious viral yields via plaque assay. As one round of Ad5-WT replication in BECs is approximately 72 hours, we first looked at 48 and 72 hours post-infection (hpi) (Fig. 2A and B). At 72 hpi, there were significantly higher viral yields in both TFII-I KO cell lines compared to the parental cells (Fig. 2B). We then performed a multistep growth curve at low multiplicity of infection to observe the effect of TFII-I KO over multiple rounds of replication. We again saw significantly higher viral titers in the TFII-I KO BECs compared to the parental cells at 3, 6, and 9 days post-infection (dpi) (Fig. 2C through E). Our data thus support the idea that TFII-I plays an inhibitory role in HAdV infection. Furthermore, in the multistep growth curve, we observed that the difference in viral titers between parental and TFII-I KO cells decreased over time as the overall amount of infectious virus increased, suggesting that HAdV sensitivity to TFII-I may be inversely proportional to the amount of virus within the cell during infection.

### TFII-I KO increases viral late protein and RNA expression in Ad5-WT infection

While the viral yield results supported an antiviral role for TFII-I in HAdV infection, we wanted to identify the earliest part of the replication cycle that was impacted by TFII-I to help determine the mechanism. We began by looking at the late stage of the HAdV replication cycle. HAdV late gene expression is dependent upon viral genome replication. All five HAdV late genes (L1, L2, L3, L4, and L5) share a common promoter called the major late promoter (MLP). Although low levels of MLP transcription are detectable prior to replication, the onset of viral DNA synthesis triggers more than a 100-fold increase in MLP expression (2). While the mechanism(s) that governs the early-to-late gene expression switch is not fully understood, it is known that MLP activation requires two intermediate viral proteins, L4-22K and IVa2, that are both expressed just prior to the onset of viral DNA replication (2). While IVa2 has its promoter, L4-22K is encoded by the late gene L4, necessitating an internal promoter, L4P, which allows L4-22K to be expressed independently of the MLP (38, 39). Interestingly, a previous study found that TFII-I was able to repress expression of L4P in the context of a transient expression assay (30). An attractive model, therefore, is that the L4P promoter is activated by the inhibition of TFII-I by E4ORF3.

**Fig 3:**
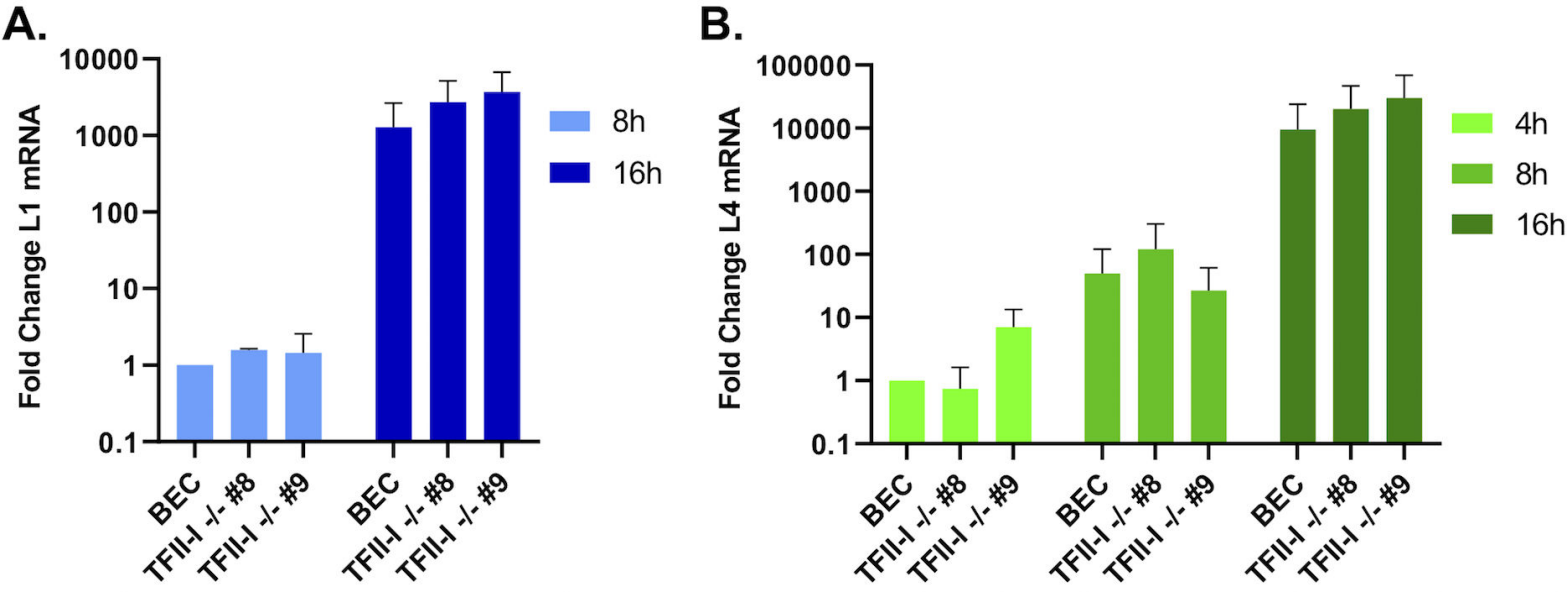
TFII-I KO does not alter viral late gene expression at early times in Ad5-WT infection. Parental and TFII-I KO BECs were infected with Ad5-WT at an MOI of 1. Total RNA was collected at the indicated times post-infection, and the levels of (**A**) L1 and (**B**) L4 mRNA were determined by RT-qPCR and normalized to GAPDH. The results shown represent the mean values ± SD; *n* = 3.

To test this, we compared the expression of viral late genes L1 and L4 over a time course of infection—if TFII-I is a specific repressor of L4P, we would expect to see transcription of L4 to begin earlier in TFII-I KO cells compared to parental BECs. BECs were infected with Ad5-WT, and RNA was collected at 4, 8, and 16 hpi. Viral RNA expression was quantified using RT-qPCR. L1 transcripts were undetectable at 4 hpi, but were detected beginning at 8 hpi (Fig. 3A). We did not observe any significant difference in L4 expression between parental and TFII-I KO cells at any of the timepoints examined (Fig. 3B). We also saw no significant difference in L1 expression between parental and TFII-I KO cells at 8 and 16 hpi. Our data are therefore not consistent with the hypothesis that TFII-I acts as a repressor of L4P at early times in the HAdV replication cycle.

**Fig 4:**
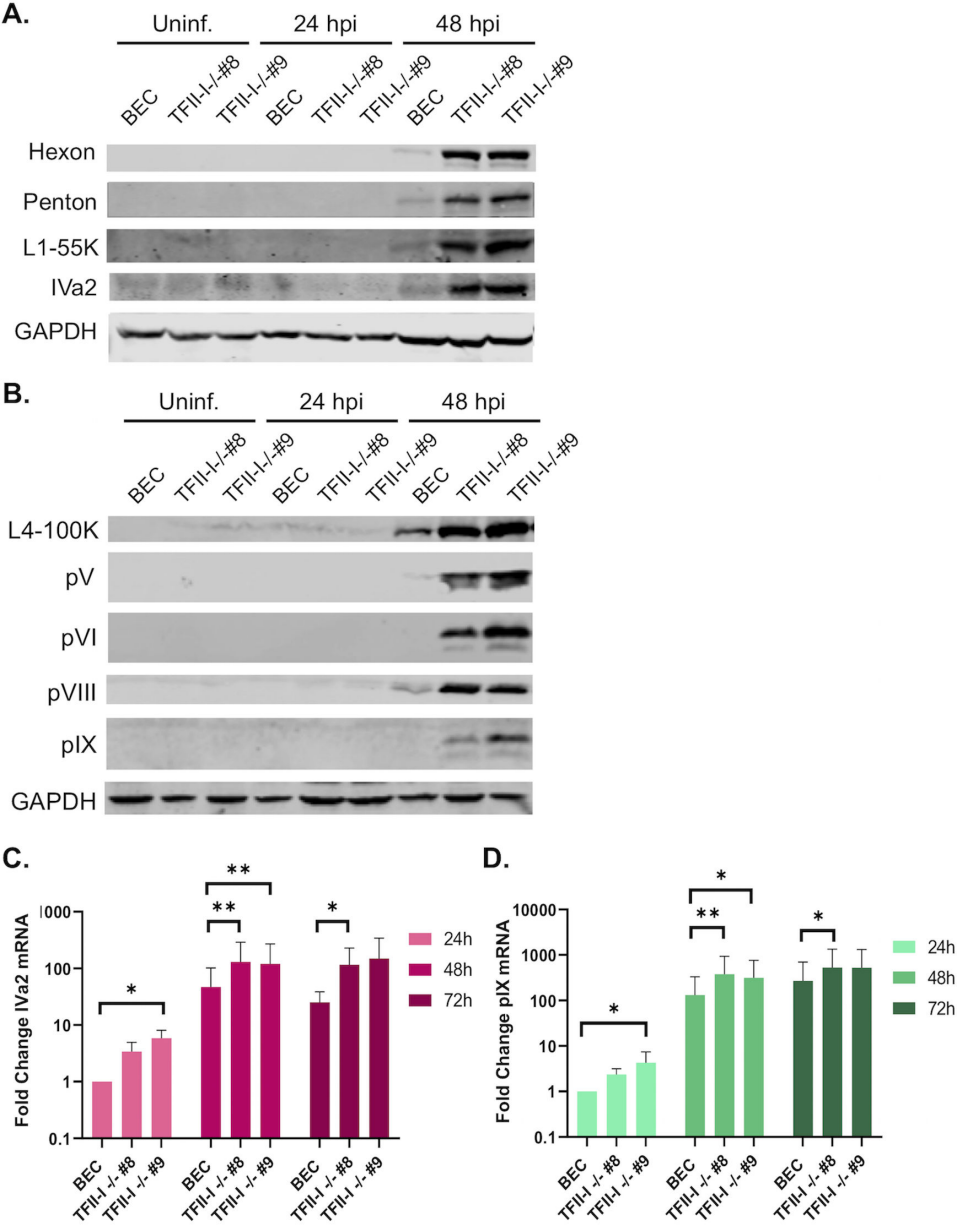
TFII-I KO increases viral late protein and intermediate stage gene expression during Ad5-WT infection. Parental and TFII-I KO BECs were infected with Ad5-WT at an MOI of 10 (**A, B**) or 1 (**C, D**). (**A, B**) Whole-cell extracts were collected at the indicated times post-infection, and the level of Ad5 intermediate and late proteins was determined by Western blot using the indicated antibodies; GAPDH was measured as a loading control. (**C, D**) Total RNA was collected at the indicated times post-infection, and the levels of IVa2 (**C**) and pIX (**D**) mRNAs were determined by RT-qPCR and normalized to GAPDH. The results shown represent the mean values ± SD; *n* = 6. * =*P* ≤ 0.05, ** =*P* ≤ 0.01, *** =*P* ≤ 0.001.

**Fig 5:**
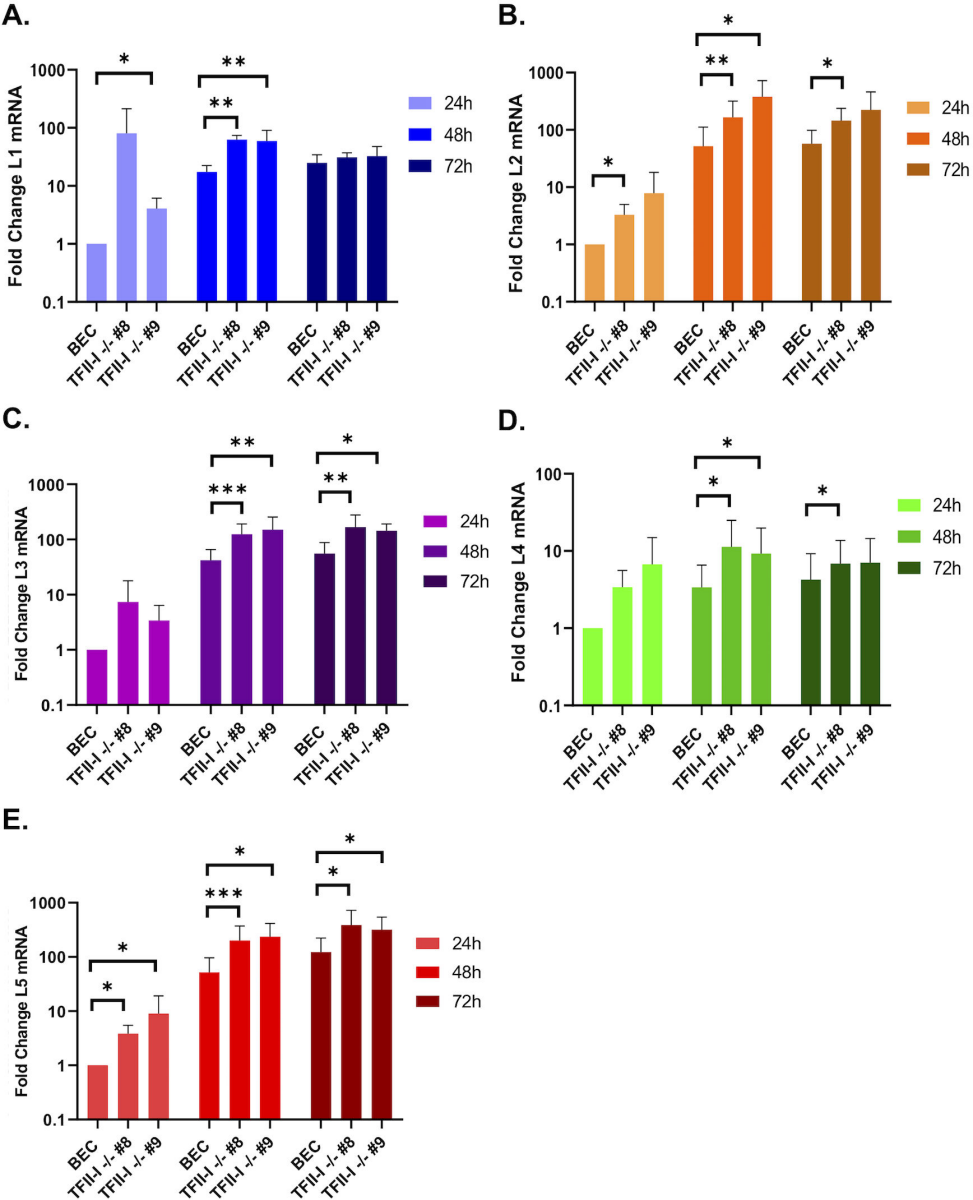
TFII-I KO increases viral late gene expression during Ad5-WT infection. Parental and TFII-I KO BECs were infected with Ad5-WT at an MOI of 1. Total RNA was collected at the indicated times post-infection, and the levels of L1–L5 mRNAs were determined by RT-qPCR and normalized to GAPDH (A, L1; B, L2; C, L3; D, L4; E, L5). The results shown represent the mean values ± SD; *n* = 6. * =*P* ≤ 0.05, ** =*P* ≤ 0.01, *** =*P* ≤ 0.001.

We next examined late protein and gene expression in parental and TFII-I KO cells. BECs were infected with Ad5-WT, and RNA and whole-cell extracts were collected at 24 and 48 hpi. Interestingly, we found that the expression of multiple viral late proteins was increased in TFII-I KO cells relative to the parental cells at 48 hpi (Fig. 4A and B). These included both major structural proteins, penton (L2) and hexon (L3), as well as packaging proteins L1-55K and IVa2 (Fig. 4A). Viral late proteins, L4-100K, pV (L2), pVI (L3), and pVIII (L4), as well as the intermediate protein pIX, were also upregulated at 48 hpi in TFII-I KO cells (Fig. 4B). The levels of viral intermediate and late gene expression were then measured by RT-qPCR. We observed significantly higher levels of the intermediate genes IVa2 and pIX (Fig. 4C and D) as well as all five late genes (Fig. 5A through E) in both TFII-I KO cell lines at 48 hpi. At 24 hpi, only L5 was consistently expressed at a statistically significantly higher level in both TFII-I KO cell lines. Together, these results show that while TFII-I does not fit the profile of a cellular repressor of L4P activity, TFII-I KO does lead to increased viral intermediate and late gene and protein expression.

### TFII-I KO transiently increases viral DNA replication in Ad5-WT infection

**Fig 6:**
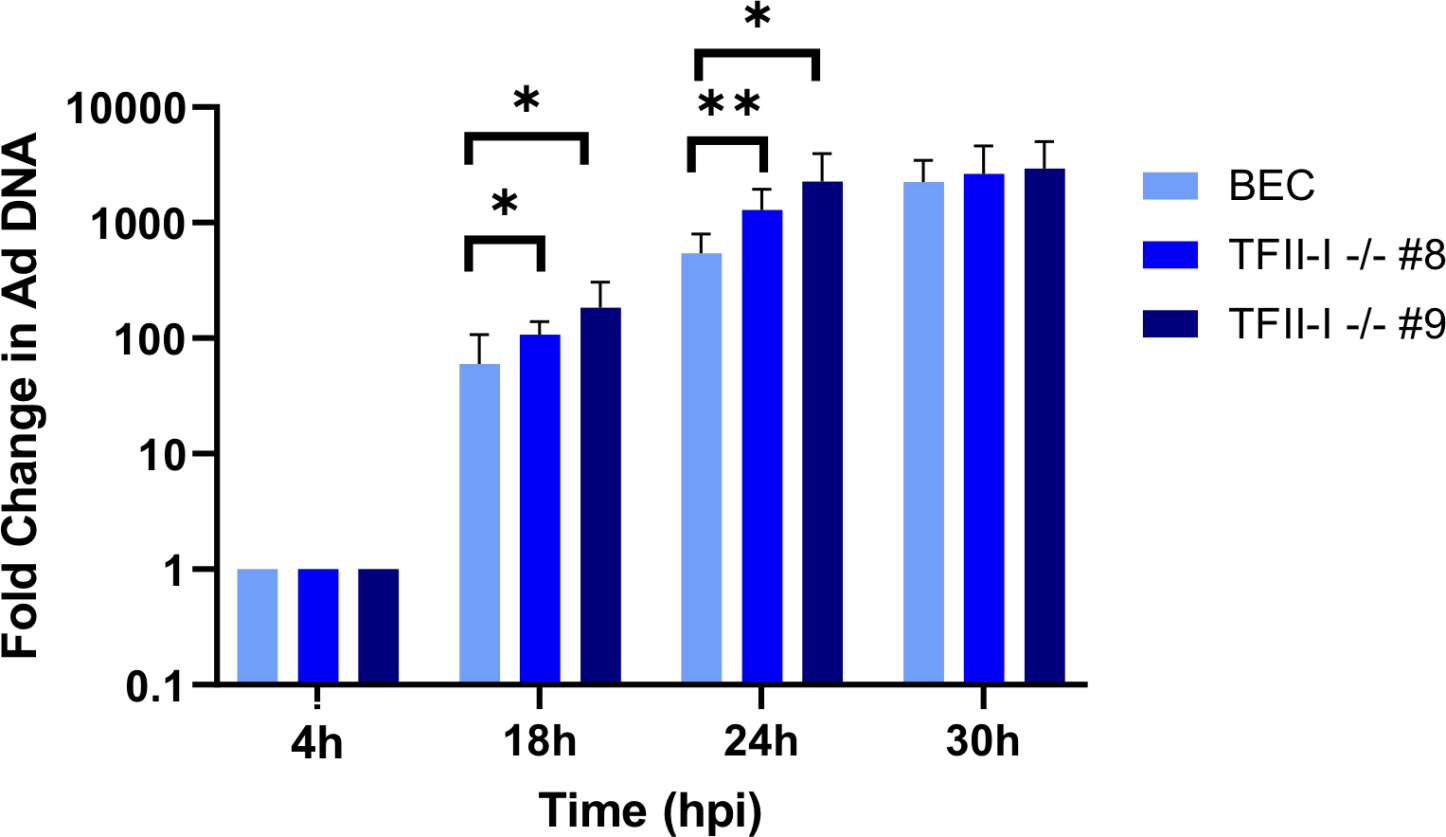
TFII-I KO transiently increases Ad5-WT viral DNA replication. Parental and TFII-I KO BECs were infected with Ad5-WT at an MOI of 1, and total DNA was isolated at the indicated time post-infection. HAdV genome levels were quantified by qPCR and normalized to endogenous GAPDH levels. The results shown represent the mean values ± SD; *n* = 6. * =*P* ≤ 0.05, ** =*P* ≤ 0.01.

We next wished to establish whether the effects of TFII-I on HAdV infection began during the late stage of infection. It is well established that Ad late gene expression is directly dependent on successful viral DNA replication (2); consequently, one potential cause of the increase in HAdV late proteins was due to an alteration in viral DNA replication that subsequently altered the onset of the late stage. To test this, we examined viral DNA replication in parental and TFII-I KO BECs. We found that at 18 and 24 hpi, viral DNA levels were significantly higher during Ad5-WT infection in both TFII-I KO cell lines compared to parental cells, but that by 30 hpi, this difference had disappeared (Fig. 6). This accords well with late RNA and protein expression in TFII-I KO cells, as we would expect DNA replication reaching peak levels earlier in infection to trigger earlier activation of late genes in TFII-I KO cells. Mechanistically, this effect could be the result of direct repression of viral DNA replication by TFII-I, or a consequence of TFII-I altering viral early gene and protein expression.

### TFII-I KO increases early protein and RNA expression in Ad5-WT infection

**Fig 7:**
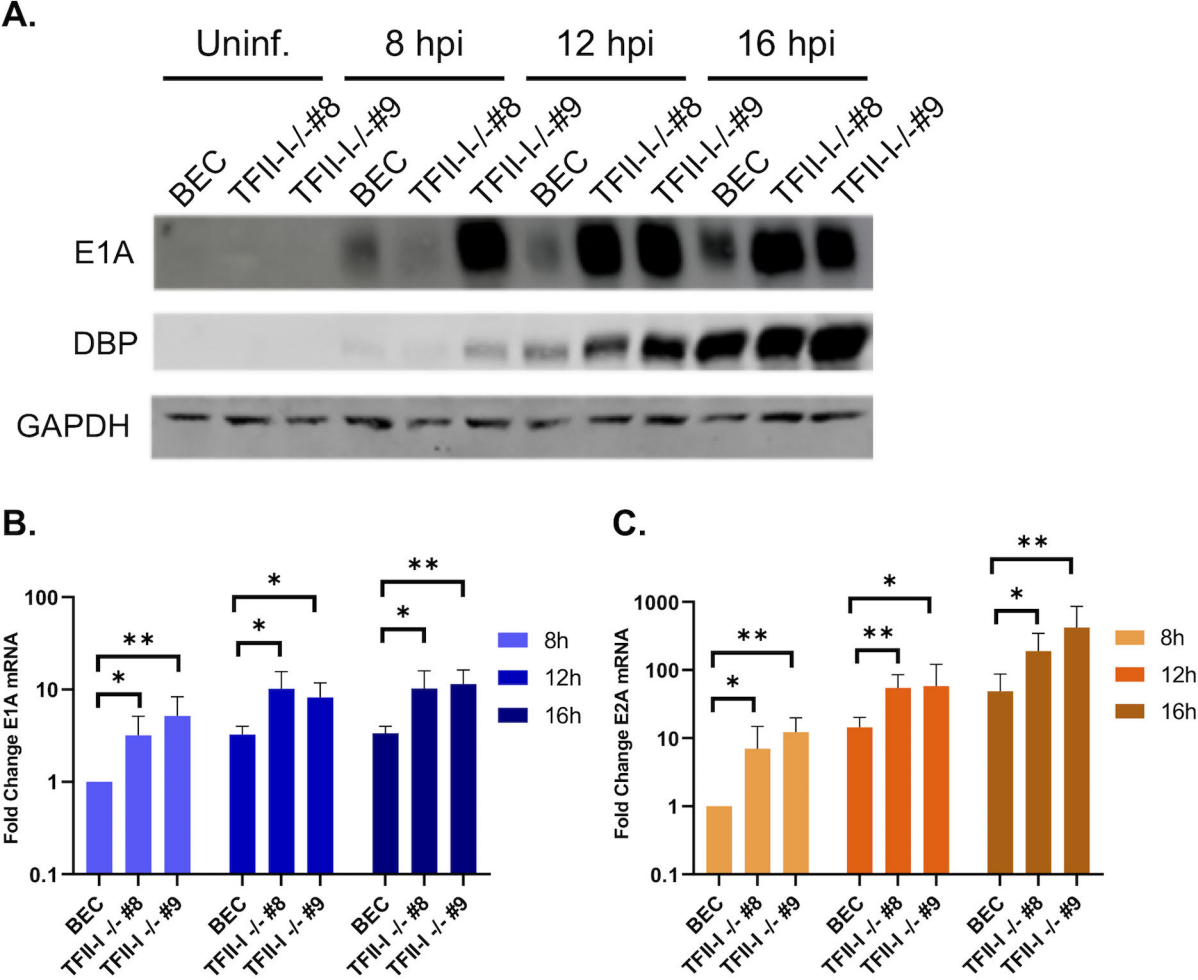
TFII-I KO increases viral early gene and protein expression during Ad5-WT infection. Parental and TFII-I KO BECs were infected with Ad5-WT at an MOI of 10 (**A**) or 1 (B, C). (**A**) Whole-cell extracts were collected at the indicated times post-infection, and the levels of E1A and DBP proteins were determined by Western blot as indicated; GAPDH was measured as a loading control. (B, C) Total RNA was collected at the indicated time post-infection, and the levels of E1A and E2A mRNAs were determined by RT-qPCR and normalized to GAPDH. The results shown represent the mean values ± SD; *n* = 6. *P ≤ 0.05, **P ≤ 0.01.

We wished to establish whether TFII-I altered Ad early gene and protein expression. We infected parental and TFII-I KO BECs with Ad5-WT and examined viral early protein and mRNA levels at 8, 12, and 16 hpi. We found that immediate-early E1A and early DBP protein levels were higher in both TFII-I KO cell lines compared to the parental BECs (Fig. 7A). This effect correlated with higher E1A (Fig. 7B) and E2A (DBP) (Fig. 7C) mRNA levels in TFII-I KO cells over the time course of infection. These results indicate that TFII-I inhibits early viral gene and protein expression, consistent with the increases in viral DNA and late gene and protein expression observed at later timepoints in TFII-I KO cells.

## DISCUSSION

We previously reported that the cellular transcription factor TFII-I is targeted for degradation by the HAdV early protein E4ORF3, a known inhibitor of the cellular antiviral response (7). Based on these studies, it was proposed that TFII-I could have an as-yet-unknown antiviral role against HAdV. Here, we show that TFII-I is capable of inhibiting Ad5 replication in normal human bronchial epithelial cells, as TFII-I KO results in higher viral yields compared to parental cells. We also show that TFII-I KO results in higher levels of viral gene and protein expression at both late and early time points in the viral replication cycle, as well as a transient increase in viral DNA replication. While many previously identified cellular targets of E4ORF3 play roles in the DDR or IFN response to HAdV (6, 8–12, 40), we show that the effect of TFII-I on Ad5 is not consistent with TFII-I acting as a part of either antiviral response. Previous studies also identified TFII-I acting to specifically repress the HAdV promoter L4P during the early stage of viral infection as a potential mechanism for TFII-I to alter viral late gene expression (30). In contrast to this hypothesis, we show that TFII-I KO has no significant effect on Ad5 late gene expression at early points in the viral replication cycle, but does significantly increase all late and intermediate gene expression later in infection. In concert with these results, we show that the expression of many Ad5 late and intermediate proteins is also increased at the same timepoints by TFII-I KO. As it is well established that HAdV late gene expression is dependent on successful viral genome replication (2), these results are consistent with the transient increase in Ad5 DNA we also observe in TFII-I prior to the onset of the late stage of infection. This increase in Ad5 DNA is complemented by the increase in Ad5 early gene and protein expression, as HAdV DNA replication is dependent on the successful completion of the early stage of infection (2). Our results are thus consistent with TFII-I acting to inhibit Ad5 replication at a much earlier stage of the viral replication cycle than was previously predicted.

It is curious that the ability of E4ORF3 to inhibit the IFN response is conserved across HAdV species, but only species C E4ORF3 targets cellular proteins involved in the DDR (12). As previous work found that only species C E4ORF3 targets TFII-I (7), we examined whether TFII-I was a member of this latter group, especially in light of TFII-I being identified as a DDR protein (27–29). However, our observations of the effect of TFII-I KO on the replication of a DDR-sensitive Ad5 mutant virus do not support TFII-I acting as a part of the DDR in HAdV infection. While more research remains to be done on species C E4ORF3 and its differences from other HAdV E4ORF3 proteins, TFII-I seems to fall into a different category of antiviral protein in HAdV infection than those previously identified, which implies that the divergence in targets cannot solely be explained by providing redundancy in DDR inhibition.

While TFII-I has previously been shown to be linked to immune signaling in the context of B and T cell receptor signaling pathways (41), it is also interesting to note that a previous study in mouse embryonic fibroblasts (MEF) found that several genes involved in the IFN response, including IRF1 and ISG15, were upregulated after TFII-I overexpression (35), while another study in HeLa cells found that TFII-I knockdown (KD) reduced expression of the IFN regulator IRF7 (23). As E4ORF3 is known to oppose the IFN response to HAdV infection (8, 9), we were interested in exploring whether TFII-I contributes to this response. However, our results did not support TFII-I KO reducing IRF7 protein expression in response to IFN signaling, or inhibiting the repression of Ad5 replication in IFN-treated cells.

One question that remains is specifically how TFII-I inhibits HAdV. TFII-I was predicted to bind the HAdV genome due to the presence of multiple Inr element consensus sequences within the viral genome (30), as well as a previous study using iPOND that found that TFII-I bound E4 mutant genomes that lacked E4ORF3 (42). Our results are consistent with at least two potential mechanisms of TFII-I inhibition of AdV gene expression. First, TFII-I may bind and specifically repress Ad5 early promoters, analogous to the repression of cellular gene expression, to reduce viral early gene expression until TFII-I is inhibited by E4ORF3, leading to subsequent reductions in viral DNA and late protein expression. Second, TFII-I may sequester cellular transcriptional activators away from viral promoter regions. Determining the exact mechanism by which TFII-I inhibits Ad5 replication will require additional studies to understand which protein-protein and protein-DNA interactions are required for the inhibitory effect. It has also been suggested that E4ORF3-mediated sequestration and degradation of TFII-I could represent a method for HAdV to modulate the function of other cellular proteins, specifically CTCF, an epigenetic regulatory protein which can be targeted to different genes by accessory proteins (42). Previous studies identified TFII-I as a key targeting factor for CTCF, particularly with respect to cellular metabolism genes (25), and as CTCF has been shown to repress gene expression in multiple DNA viruses, including EBV and KSHV (43), it has been proposed that TFII-I binding to the HAdV genome during infection might recruit CTCF to HAdV promoters to repress them (42). However, a recent study on the effect of CTCF knockdown on Ad5 replication found that reductions in CTCF levels led to a decrease in viral DNA replication and late gene expression, and that CTCF bound the Ad genome only at a few specific regions in a replication-dependent manner (44). By contrast, we observed an increase in viral DNA and late gene expression as well as increased expression of Ad5 early genes at times prior to Ad5 genome replication. While more work is needed to better understand the role of CTCF in HAdV infection, what is known does not seem to support a role for TFII-I acting to target CTCF to the HAdV genome, or for a hypothetical disruption of CTCF binding to the viral genome in the absence of TFII-I being of benefit to HAdV.

While our work focuses solely on the role of TFII-I in HAdV infection, it is interesting to consider whether TFII-I may also inhibit other DNA viruses. Outside HAdV, TFII-I has only been shown to play a role with retroviruses, specifically HIV and RSV, by increasing expression of the retroviral provirus (45–48). However, a previous study using iPOND found that TFII-I bound the HSV-1 genome in infected cells, suggesting that TFII-I may also play a role in HSV infection (49). While more work would be required to establish whether or not the interaction between TFII-I and HSV-1 genomes has a functional consequence, if found, it would suggest that TFII-I might possess a more general role in modulating DNA virus infections as opposed to a unique role in HAdV infection. The fact that many DNA viral genomes contain Inr elements and E-box consensus sequences at least leaves open the possibility for TFII-I binding, although additional studies will be needed to establish whether this occurs in other DNA viruses beyond HAdV, and whether such interactions lead to functional consequences. In conclusion, we have shown that TFII-I KO enhances Ad5 replication, in agreement with previous research that suggested that TFII-I acts to inhibit HAdV infection. We have also shown that the effects of TFII-I KO are observable beginning very early in infection, contrary to previous predictions that TFII-I would likely specifically repress HAdV late gene expression. While more work needs to be done to establish the exact mechanism, our results are consistent with a model where TFII-I reduces Ad5 early gene expression by binding the viral genome during infection.

## MATERIALS AND METHODS

### Cell culture and viruses

Normal human diploid fibroblasts immortalized by the expression of human telomerase (HDF-TERT) (50), Nijmegen breakage syndrome (NBS) (34), and HEK293 cells were maintained in Dulbecco’s modified Eagle’s medium (DMEM) with 10% fetal bovine serum (Life Technologies) supplemented with 100 µg/mL penicillin and streptomycin. N52.E6-Cre cells were maintained in DMEM containing 10% Fetal Clone III serum (Hyclone). N52.E6-Cre cells, a gift from G. Schiedner and S. Kochanek, Center for Molecular Medicine (ZMMK), University of Ulm, Ulm, Germany, were derived from the E1-expressing cell line N52.E6 (51). Cre recombinase was not important for these experiments. Human bronchial epithelial cells (HBEC3-KT, ATCC) were maintained in airway epithelial cell media supplemented with the Epithelial Cell Growth kit as per the manufacturer’s instructions (ATCC). The following viruses were used in these studies: wild-type adenovirus type 5 (HAdV-C5, Ad5-WT) and dl355/inORF3 (E4-ORF3^-^/E4-ORF6^-^) (19). Virus infections were performed for 1 h at 37°C at the multiplicities of infection (MOI) specified in the figure legends, followed by the removal of virus and replacement with fresh medium. BECs and HDF-TERT cells were treated with IFNa or IFNg (PBL Assay Sciences) at 500 U/mL or 1,000 U/mL, respectively. For HBEC3-KT and HDF-TERT cells, an MOI of 25 virus particles/cell (p/cell) results in infection of ~20% and ~10% of the cell population, respectively, as previously determined (52).

### Viral replication assay and RT-qPCR

Total cellular DNA was purified at the specified timepoints using a Qiagen DNeasy blood and tissue kit. The number of viral and cellular genome copies was determined by qPCR using primer pairs that recognize either the HAdV-C5 genome or cellular glyceraldehyde-3-phosphate dehydrogenase (GAPDH) gene using DyNAmo HS SYBR green qPCR Kit (Thermo). The viral DNA copy numbers were normalized to GAPDH, and then the fold increase in viral copy numbers was calculated by normalizing the amount of DNA present at later timepoints to a 4 or 6 hpi input timepoint. For RT-qPCR, the cells were infected for the specified time post-infection, and total cellular RNA was isolated using a Qiagen RNeasy kit. Equal amounts of total RNA were used to synthesize the first strand cDNA using either RNA to cDNA EcoDry Premix (Takara Bio) or a SuperScript II Reverse Transcriptase and oligo-dT primer (Life Technologies). Equal amounts of cDNA were then subjected to qPCR using primer pairs that recognize individual viral mRNAs and cellular GAPDH mRNA. Viral mRNAs were normalized to the internal control GAPDH mRNA. Oligonucleotides used to measure viral DNA replication were as follows: HAdV-C5 ITR and E1A enhancer (GCGAAAATGGCCAAATGTTA and TAATGAGGGGGTGGAGTTTG) and GAPDH (GCCATGTAGACCCCTTGAAGAG and ACTGGTTGAGCACAGGGTACTTTAT). Oligonucleotides used to measure viral mRNA levels were as follows: L1 (GGCGCAGCTGTTCCTTATAG and GGGAACGTAAGGGGTATGGT), L2 (CAACAAGTCAACGGATGTGG and TCGTTCACATTTGGCATGTT), L3 (GCACCTATGACAAGCGCTTT and GGTAAACCTGCTTGAGTCGC), L4 (CACCTGCTGTGCACTTCCTA and TAGGTTGCAGCGACAGTGAC), L5 (GGAAATATCTGCACCCCTCA and GCAGGGCTAGCTTTCCTTCT), IVa2 (GTGTCATAGTCCAGCCCCTC and CGGAATCGGTATTTCTCGCG), pIX (GCCCGCAAACTCTACTACCT and AATTGTGCCAAAAGAGCCGT), E1A (CGACTCTGTAATGTTGGCGG and CTCGTGGCAGGTAAGATCGA), and E2A (GGCCAAGATCGTGAAGAACC and AAAAGCCACCTGAGCCTTTG).

### Western blot analysis

Whole-cell lysates were prepared by suspending cell pellets in SDS lysis buffer (50 mM Tris-HCl, pH 6.8, 4% SDS) and boiling for 10 min. A Pierce BCA Protein assay kit was used to determine protein concentration. Equal amounts of all proteins were run on an SDS-PAGE gel, followed by transfer to a nitrocellulose membrane. The membranes were blocked in Tris-HCl-buffered-saline (TBS) containing 3% BSA for 1 h, rocking at room temperature or overnight at 4°C. After blocking, the membranes were incubated rocking at 4°C overnight with primary antibodies against the protein of interest. Membranes were washed three times with TBS buffer containing 0.1% Tween 20 (TBS-T) and then incubated with either IRDye 800CW-conjugated goat anti-rabbit antibody (926-32211, Li-COR, 1:5,000) or IRDye 680RD-conjugated goat anti-mouse antibody (925-068071, Li-COR, 1:5,000) for 1 h at room temperature. After three washes with TBS-T buffer and rinsing with TBS, the ODYSSEY CLx infrared imaging system (Li-COR) was used to capture images. Alternatively, HRP-conjugated antibodies (Amersham) were used in conjunction with ECL Western blotting (Millipore Immobilon or ThermoScientific), and the resulting images were captured using a GE ImageQuant LAS 500. The antibodies used were as follows: rabbit polyclonal TFII-I antibody (Cell Signaling Technologies) (1:1,000 dilution), rabbit polyclonal IRF7 (Cell Signaling Technologies) (1:1,000), mouse monoclonal hexon antibody (MAB0850; Abnova) (1:5,000 dilution), rabbit polyclonal penton antibody (gift from Carl Anderson, Brookhaven National Laboratory) (1:1,000 dilution), rabbit polyclonal IVa2 antibody (53) (1:1,000 dilution), rabbit polyclonal L1-52/55K antibody (53) (1:1,000 dilution), rabbit polyclonal V antibody (gift from David Matthews, University of Bristol) (1:1,000 dilution), rabbit polyclonal L4-100K antibody (54) (1:1,000 dilution), rabbit polyclonal VIII antibody (gift from Ann Tollefson and William Wold, St. Louis University) (1:400 dilution), rabbit polyclonal E1A antibody (SC430; Santa Cruz Biotechnology) (1:500 dilution), rabbit polyclonal DNA binding protein (DBP) antibody (gift from Peter van der Vleit, University Utrecht, Netherlands) (1:1,000 dilution), mouse monoclonal α-Tubulin (Sigma-Millipore #T5168) (1:10,000), rabbit polyclonal GAPDH (Millipore Sigma Aldrich) (1:7,500), and rabbit monoclonal vimentin (NeoMarkers) (1:2,000).

### Generation of TFII-I CRISPR-Cas9 knockout cell lines

pLenti-CRISPR-v2 (Addgene plasmid #52961) vectors containing puromycin or blasticidin resistance genes were engineered to express 20 nt targeting sequence for TFII-I (TTGCAAGATCATAGTTCTCG). Individual colonies were isolated following Lenti-CRISPR transduction and selection and then screened by western blot for gene knockout.

### Plaque assays and immunofluorescence analysis

Cell lysates were prepared by collecting infected cells and culture supernatants, which were then lysed by four cycles of freeze-thawing. Titers were determined using plaque assay in 293 or N52.E6-Cre cells. Briefly, 293 or N52.E6-Cre cells were infected with two dilutions of each lysate and overlaid at 1 h post-infection. Plaques were counted at 8 and 10 days post-infection and used to calculate PFU/mL.

### Statistical analysis

All numerical values represent the mean ± standard deviation. For Western blots, a representative replicate is shown for each experiment. Statistical significance was calculated using a paired *t*-test; for RT-qPCR data, the data were first normalized by log-normal transformation.

## Data Availability

All of the data and methods are presented within the article.
